# *Vaccinium bracteatum* Leaf Extract Reverses Chronic Restraint Stress-Induced Depression-Like Behavior in Mice: Regulation of Hypothalamic-Pituitary-Adrenal Axis, Serotonin Turnover Systems, and ERK/Akt Phosphorylation

**DOI:** 10.3389/fphar.2018.00604

**Published:** 2018-07-09

**Authors:** Dool-Ri Oh, Ji-Seok Yoo, Yujin Kim, Huwon Kang, Hunmi Lee, So J. Lm, Eun-jin Choi, Myung-A Jung, Donghyuck Bae, Kyo-Nyeo Oh, Ji-Ae Hong, Ara Jo, Jawon Shin, Jaeyong Kim, Young R. Kim, Seung S. Cho, Beom-Jin Lee, Chul yung Choi

**Affiliations:** ^1^Jeonnam Bioindustry Foundation, Jeonnam Institute of Natural Resources Research, Jeollanamdo, South Korea; ^2^College of Pharmacy and Research Institute of Drug Development, Chonnam National University, Gwangju, South Korea; ^3^Bioavailability Control Laboratory, College of Pharmacy, Ajou University, Suwon, South Korea; ^4^Department of Pharmacy, College of Pharmacy, Mokpo National University, Muan, South Korea

**Keywords:** *Vaccinium bracteatum* Thunb., antidepressants, neuroprotection, chronic restraint stress, corticosterone, HPA axis, serotonin turnover

## Abstract

The leaves of *Vaccinium bracteatum* Thunb. are a source of traditional herbal medicines found in East Asia. The present study aimed to evaluate the mechanisms underlying the antidepressant-like effects of water extract of *V. bracteatum* Thunb. leaves (VBLW) in a mouse model of chronic restraint stress (CRS) and to identify the possible molecular *in vitro* mechanisms of the neuroprotective effects. The CRS-exposed mice were orally administered VBLW (100 and 200 mg/kg) daily for 21 days consecutively. The behavioral effects of VBLW were assessed through the forced swim test (FST) and the open field test (OFT). The levels of serum corticosterone (CORT), corticotropin releasing hormone (CRH), and adrenocorticotropin hormone (ACTH), brain monoamines, such as serotonin, dopamine, and norepinephrine, and serotonin turnover by tryptophan hydroxylase 2 (TPH2), serotonin reuptake (SERT), and monoamine oxidase A (MAO-A) were evaluated, in addition to the extracellular signal-regulated kinases (ERKs)/protein kinase B (Akt) signaling pathway. CRS-exposed mice treated with VBLW (100 and 200 mg/kg) showed significantly reduced immobility time and increased swimming and climbing times in the FST, and increased locomotor activity in the OFT. Moreover, CRS mice treated with VBLW exhibited significantly decreased CORT and ACTH, but enhanced brain monoamine neurotransmitters. In addition, CRS mice treated with VBLW had dramatically decreased protein levels of MAO-A and SERT, but increased TPH2 protein levels in the hippocampus and the PFC. Similarly, VBLW significantly upregulated the ERKs/Akt signaling pathway in the hippocampus and the PFC. Furthermore, VBLW showed neuroprotective effects via increased CREB phosphorylation in CORT-induced cell injury that were mediated through the ERK/Akt/mTOR signaling pathways. These results suggested that the antidepressant-like effects of VBLW might be mediated by the regulation of the HPA axis, glucocorticoids, and serotonin turnover, such as TPH2, SERT, and MAO-A, as well as the concentration of monoamine neurotransmitters, and the activities of ERK and Akt phosphorylation, which were possibly associated with neuroprotective effects.

## Introduction

Depression is a common mental disorder. The World Health Organization (WHO) reported that depression will provide the greatest contribution to the global disease burden by the year 2030 (Cohn et al., 2012). Depressive disorders are characterized by changes in mental status induced by hyperactivity of the HPA axis and neurotrophin dysfunction (Radley et al., 2004; Angelucci et al., 2005). Stress is known to be a risk factor for the development of major depression. Stress models (e.g., restraint stress and unpredictable mild stress) is common in animal studies to mimic the development of depressive-like symptoms, such as altered weight gain, changed of the physical state, cognitive deficits and locomotor activity deficit. The restraint stress models has two known conditions, acute (30 min for 1 day) and chronic (6 h per day for 21 days), widely used physical stressors (Chattarji et al., 2015). Of these, CRS model of developing clinical depression, are associated with the dysregulation of the HPA axis (Chiba et al., 2012). CRS may activate the HPA axis, which includes a feedback loop composed of the hypothalamus, pituitary, and adrenal glands, which is thought to be closely related to the inhibition of negative feedback by endogenous hormones, such as CRH, ACTH, and CORT (Chang et al., 2015; Franco et al., 2016).

Previous studies have revealed that the depletion of monoamines, such as serotonin, dopamine, and norepinephrine, might be an important mechanism underlying depression, which is one of the most widely accepted hypotheses (Belmaker and Agam, 2008; Mahar et al., 2014).

Various early studies indicated that the levels of monoamines in the brain regions, such as the hippocampus and prefrontal cortex (PFC), were increased after treatment with antidepressants (Bhagya et al., 2011; Blier, 2016). In addition, many studies have demonstrated the association of CRS with structural degeneration and the impaired functionality of the hippocampus and the PFC (Mah et al., 2016). Depression and chronic stress disrupt BDNF signaling, including reductions in the ERKs, PI3K/Akt, and CREB pathways, which are important mediators of the signal transduction pathways (Tomita et al., 2013; Plattner et al., 2015).

Moreover, CRS affects the molecular mechanisms of catecholaminergic turnover in the brain (Popović et al., 2017). It is important to examine the expression of key enzymes involved in catecholamine biosynthesis, reuptake, and degradation (Kvetnansky et al., 2009). Among the catecholamines, such as serotonin, dopamine, and norepinephrine, serotonin turnover (i.e., biosynthesis, reuptake, and degradation) is critically associated in patients with MDD. It has been well established that serotonin modulates the stress response through interaction with the HPA axis (Chen and Miller, 2013). Thus, in the brain, antidepressants may act to modulate serotonin turnover, which has also been associated with TPH (serotonin synthesis), SERT (reuptake), and monoamine oxidase (MAO; degradation).

*Vaccinium bracteatum* Thunb. of the family *Ericaceae*, is and evergreen shrub or small tree found in East Asia, especially in China and Korea. The leaves are used as a traditional herbal medicine, to stain cooked rice, and as a natural dye for protein, hair, and starch (Wang and Yao, 2006). Previous studies reported that the leaves of *V. bracteatum* had anti-oxidant, anti-inflammatory anti-fatigue, anti-microbial, and anti-diabetic activities, and protected the retina from light damage (Wang et al., 2006, 2008, 2010, 2011, 2013; Kwon et al., 2016). In addition, it has also been reported that the leaves of *V. bracteatum* leaves contain flavonoids, polyphenols, and diterpenes. The constituent flavonoids, such as orientin, vitexin, chrysin, apigenin, and kaempferol, are known to have antioxidant activity; in addition, some flavonoids have anxiolytic properties (Budzianowski et al., 1991; Wang et al., 2011).

We previously reported that the efficacy of the antidepressant-like effects of *V. bracteatum* fruit extract might be mediated through the regulation of monoaminergic systems and glucocorticoids, which is possibly associated with neuroprotective effects and the antagonism of the 5-HT_2A_ receptor (Oh et al., 2018). However, the molecular and cellular mechanisms underlying the antidepressant-like effects of *V. bracteatum* leaves remain unclear.

In the present study, we first evaluated the involvement of the neuroprotective effects in the CORT-induced cytotoxicity of *V. bracteatum* leaves (VBL) extract after treatment with a high concentration of CORT in SH-SY5Y cells, which indicated that successful replication of *in vitro* conditions of stress-depression. Moreover, the antidepressant-like effects of VBL water extract (VBLW) on the FST, the OFT, and the levels of monoamine neurotransmitters (e.g., serotonin, dopamine, and norepinephrine) and endogenous hormones (e.g., CRH, ACTH, and CORT) associated with the monoamine system and HPA axis in the brain (hippocampus and PFC) and serum in CRS-induced depression mice were studied. We also investigated the underlying mechanisms of the action associated with catecholaminergic turnover systems (particularly the serotonergic system) in CRS-exposed mice, including the protein expression of TPH2 (serotonin synthesis), SERT, and MAO-A (degradation). We also evaluated the phosphorylation of the ERK/Akt pathways in the hippocampus and the PFC. In addition, we evaluated the effects of five organic solvent fractions isolated from VBL on CORT-induced cell injury by using a cell viability assay.

## Materials and Methods

### Chemicals and Reagents

Minimum Essential Medium and FBS were purchased from Invitrogen Inc. (Grand Island, NY, United States). CORT, MTT, PMSF, DTT, aprotinin, leupeptin, BSA, orientin (97% purity), isoorientin (98% purity), and chlorogenic acid (95% purity) were purchased from Sigma Chemical Co. (St. Louis, MO, United States). Serotonin, norepinephrine, dopamine, and CORT ELISA kits were purchased from Abnova Corp. (Taipei City, Taiwan). CRH and adrenocorticotropin hormone (ACTH) ELISA kits were purchased from USCN Life Science (Wuhan, Hubei, China). Anti-p-CREB, anti-CREB, and HRP-conjugated anti-rabbit IgG antibodies were purchased from Cell Signaling Technology (Beverly, MA, United States). Anti-tryptophan hydroxylase 2 (TPH2) and monoamine oxidase (MAO) were purchased from Abcam Inc. (Cambridge, MA, United States). Serotonin transporter (SERT) was purchased from EMD Millipore (Billerica, MA, United States). Wortmannin, rapamycin, H89, and PD98059 were purchased from Calbiochem (San Diego, CA, United States).

### Preparation of the Extract

The VBL (specimen voucher number: JINR-VBL006) used in this study were collected in Goheung county (Jeollabukdo, Republic of Korea), authenticated by Dr. Choi at the Jeollanamdo Institute of Natural Resources Research (JINR), Jangheung, Jeollanamdo, South Korea, and extracted in 20 volumes of water at 100°C for 4 h. The extracted solution was filtered, concentrated with an evaporator under a vacuum, and freeze-dried. Finally, the dried water extract (VBLW) was stored at 4°C until assayed.

### Isolation of VBLW by Activity-Guided Fractionation

The dried crude extract of VBLW (60 g) was suspended in water and fractionated successively with *n*-hexane (3 × 1000 mL), chloroform (CHCl_3_, 3 × 1000 mL), ethyl acetate (EtOAc, 3 × 1000 mL), and *n*-butanol (BuOH, 3 × 1000 mL).

### Standardization of VBLW

**FIGURE 1:**
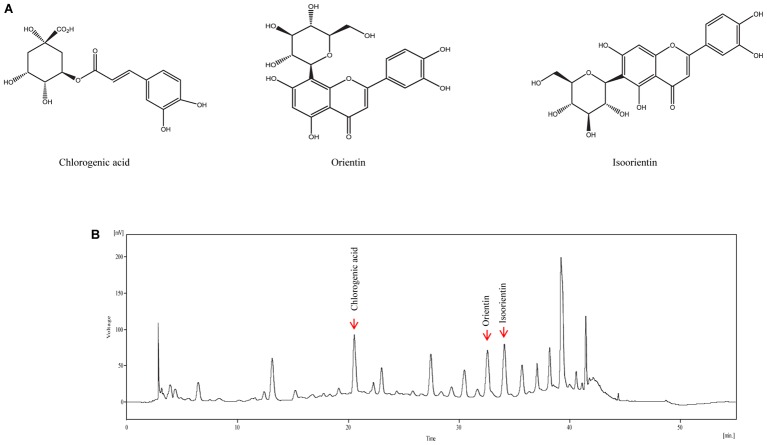
Representative high-performance liquid chromatography (HPLC) chromatogram of VBLW. **(A)** Structural formulas of the three compounds. **(B)** Mobile phase A was methanol and mobile phase B was water (containing 0.1% formic acid) and the following elution profile was used: Initial, 15% A; 5–10 min, 15–20% A; 10–15 min, 20–30% A; 15–30 min, 30–40% A; 30–37 min, 40–60% A; 37–40 min, 60–100% A; 40–45 min 100% A; 45–50 min, 100–15% A; 50–55 min, 15% A. The flow rate was 1 mL/min, the injection volume was 20 μL, and the detection wavelength was 254 nm. In the present case, the detection of chlorogenic acid, orientin, and isoorientin occurred at approximately 20.55, 32.56, and 34.09 min, respectively. The concentration of chlorogenic acid, orientin, and isoorientin content in VBLW was approximately 7.06 mg/g, 4.87 mg/g, and 8.17 mg/g, respectively.

The analysis was performed by using a YL 9110 HPLC system (Young Lin Instrument Co. Ltd., Seoul, Republic of Korea) comprising a binary pump (YL9111), a UV/Vis detector (9120), and an auto sampler (9150). The column was a Pack pro-C18 (250 mm × 4.6 mm, 5 μm, YMC, Japan) and the detection wavelength was set at 254 nm for the water extract. The column thermostat was maintained at 35°C. Mobile phase A was methanol and mobile phase B was water containing 0.1% formic acid; the following elution profile was used: initial, 15% A; 5–10 min, 15–20% A; 10–15 min, 20–30% A; 15–30 min, 30–40% A; 30–37 min, 40–60% A; 37–40 min, 60–100% A; 40–45 min 100% A; 45–50 min, 100–15% A; 50–55 min, 15% A (**Figure 1**). The flow rate was 1 mL/min and the injection volume was 20 μL.

### *In Vitro* Experiments

#### SH-SY5Y Cell Cultures

The SH-SY5Y human neuroblastoma cell line was purchased from the American Type Culture Collection (ATCC, CRL-2266, Manassas, VA, United States) and cultured in MEM supplemented with 10% FBS, 100 U/mL penicillin, and 100 μg/mL streptomycin at 37°C in a humidified atmosphere containing 5% CO_2_.

#### Cell Viability Assay

Cell viability was assessed by using the MTT assay. SH-SY5Y cells were seeded at a density of 1.5 × 10^4^ cells/well in a 96-well plate for 24 h and exposed to various concentrations of VBLW and CORT for 24 h. To evaluate the neuroprotective effect of VBLW against CORT-induced cell injury, the cells were treated with CORT (1 mM) for 24 h in the absence or presence of VBLW and the organic solvent fractions at the indicated concentrations 2 h prior to CORT treatment. In experiments involving kinase inhibitors, the cells were treated with various inhibitors, such as wortmannin (PI3K inhibitor, 200 nM), rapamycin (mTOR inhibitor, 10 μM), H89 (PKA inhibitor, 10 μM), or PD98059 (ERKs inhibitor, 10 μM) 1 h prior to VBLW treatment. At the end of the treatment, MTT solution (5 mg/mL; 20 μL/well) was added to each well and incubated for 4 h. Subsequently, the supernatants were removed and the formazan crystals were solubilized in 150 μL dimethyl sulfoxide. The OD was determined at 540 nm.

### *In Vivo* Experiments

#### Animals

Five-week-old male ICR mice (weight: 23–28 g) were purchased from Samtako Bio Korea (Osan, Republic of Korea). The animals were maintained at a constant room temperature of 22 ± 2°C with a humidity of 50 ± 5%, given free access to water and food, and a 12/12 h light:dark cycle (lights on at 8:00 am). The animals were acclimatized for 7 days prior to the start of the experiments. All experiments were approved by the Institutional Animal Care and Use Committee (IACUC) at Jeonnam Institute of Natural Resources Research (approval no. JINR-1608-2016). All animal experiments were conducted in accordance with the IACUC guidelines.

#### Drug Administration and Experimental Groups

The mice were randomly assigned to five groups (*n* = 5 per group): Group I received vehicle (saline) and served as the control; Group II was subjected to CRS and received vehicle (CRS + saline); Group III was subjected to CRS and received escitalopram oxalate 10 mg/kg/day (CRS + EO 10); Group IV was subjected to CRS and received VBLW 100 mg/kg/day (CRS + VBLW 100); Group V was subjected to CRS and received VBLW 200 mg/kg/day (CRS + VBLW 200). All drugs and vehicle were administered orally using a sonde needle at 10:30 a.m. once per day for 21 consecutive days. CRS was performed once daily between 11:00 and 5:00 p.m. for 21 consecutive days. The body weight and food intake were measured twice per week. Food intake data was expressed using intakes food weight per day per mice. EO, a known selective SERT inhibitors (SSRI) antidepressants drug, was used as the positive control (Park et al., 2014).

#### Chronic Restraint Stress Procedure

Thirty minutes after drug administration, CRS was applied for a period of 6 h by placing the animal in clear plastic tubes (7 cm diameter × 20 cm length) adjusted to the animal’s size, under a 60 W light for 21 consecutive days in accordance with a previously described method with minor modifications (Chiba et al., 2012). During restraint stress, the animals were not physically compressed, but were deprived of food and water. After the last restraint session, mice were evaluated by using the OFT and FST behavioral tests, consecutively.

#### Behavioral Evaluation

##### Open field test

General locomotor activity was evaluated by using the OFT. The mice were tested immediately before the FST. Thirty minutes after the final drug administration, the mice were individually placed into a wooden box. The OFT apparatus consisted of a 60 × 60 × 20 cm wooden box divided into 25 equal squares. Each mouse was gently placed in a corner of the apparatus and observed for 5 min; one count was made when the mouse completely crossed from one square to the next, in accordance with the method described previously (Oh et al., 2018). After each trial, the apparatus was cleaned with 70% ethanol.

##### Forced swim test

The FST was conducted in mice in accordance with previous reports with slight modification (Moreau et al., 2008; Xiong et al., 2011). The mice were individually placed in a plexiglass cylinder (diameter 15 cm) filled with 20 cm of water at 22–25°C. The mouse was exposed to 15 min of FST (training session). After 24 h, the animals were forced to swim for a 6 min test session. The duration of immobility, climbing, and swimming behaviors during the last 5 min of the test was recorded following the method described previously (Oh et al., 2018).

### Biochemical Analysis

#### Blood and Tissue Sampling

The mice were sacrificed immediately after the behavioral experiments and blood samples were collected during decapitation. The serum was separated by using refrigerated centrifugation at 3,000 rpm and 4°C for 20 min and stored at -80°C until further analysis. The brains were quickly removed, the hippocampus and PFC were rapidly isolated, immediately frozen in liquid nitrogen, and stored at -80°C until further analysis.

#### Measurement of Serum Biochemical Parameters

Serum concentrations of glutamic oxaloacetic transaminase (GOT), glutamic pyruvic transaminase (GPT), and blood urea nitrogen (BUN) levels were measured using the appropriate kits (DRICHEM4000i, FUGI-FILM, Tokyo, Japan).

#### Measurement of Serum CORT, CRH, and ACTH Levels

The CORT, CRH, and ACTH in serum were determined by ELISA in accordance with the manufacturer’s instructions (CORT, Abnova; CRH and ACTH, USCN Life Science). The results were expressed in pg/mL.

#### Measurement of Brain Monoamine Neurotransmitter Levels

The quantification of monoamines was performed in accordance with the method described previously (Lee et al., 2010) with some modifications. Briefly, the hippocampus and PFC were homogenized in 10 volumes of an extract buffer (PRO-PREP^TM^ protein extraction solution, iNtRON Biotechnology, Sungnam, Korea) on ice and incubated for 2 h at 4 °C with shaking. Subsequently, the lysates were centrifuged separately at 13,000 rpm for 20 min at 4°C. Protein contents were determined by the BCA protein assay reagent (Thermo Scientific, Rockford, IL, United States) with BSA used as the standard. The monoamines, serotonin, norepinephrine, and dopamine were determined in the homogenates of the hippocampus and PFC as per the method described by the manufacturer (Abnova). The results were expressed as pg/mL of serotonin, norepinephrine, and dopamine levels.

#### Western Blot Analysis

The hippocampus and PFC were thawed at room temperature. Homogenization was performed in PRO-PREP^TM^ Protein Extraction Solution (iNtRON Biotechnology) at 4°C for 2 h and centrifuged at 13,000 rpm for 20 min in a cooled centrifuge. The supernatants were collected and used to quantify the total protein.

SH-SY5Y cells were seeded in a 6-well plate at a density of 2 × 10^5^ cells/well for 24 h. The cells were pre-treated with VBLW at the indicated concentrations at 24 h prior to CORT treatment and exposed to CORT (1 mM) for 12 h. The nuclear fractions were prepared in accordance with a previously reported procedure (Abmayr et al., 2006), with some modifications. The cells were rinsed with ice-cold PBS and resuspended in lysis buffer A (10 mM HEPES [pH 7.9], 10 mM KCl, 2 mM MgCl_2_, 0.1 mM EDTA, 0.1 mM PMSF, 1 mM DTT, 2 μg aprotinin, and 2 μg leupeptin). The cells were allowed to swell on ice for 10 min. Subsequently, the lysates were sheared several times through a 21-gauge needle, after which 0.5% Triton X-100 was added. After 20 min on ice, the lysate was centrifuged at 3,000 rpm for 5 min in a cooled centrifuge. Then, the nuclear pellet was resuspended in lysis buffer B (20 mM HEPES [pH 7.9], 50 mM KCl, 300 mM NaCl, 1 mM EDTA, 10% glycerol, 0.1 mM PMSF, 2 μg aprotinin, and 2 μg leupeptin), incubated on ice for 10 min, and centrifuged at 14,000 rpm for 5 min. The supernatant (the nuclear fraction) was used in subsequent assays; the samples were either used immediately or stored at -80°C until assayed.

Protein content was determined by the BCA protein assay reagent using BSA as a standard. Protein lysates (brain tissue lysate, 70 μg/well; cell lysate, 50 μg/well) were separated in 10% SDS-PAGE gels by using Power Pac Basic electrophoresis apparatus (Bio-Rad, Hercules, CA, United States). The protein samples were transferred to a PVDF membrane (0.45 mm pore size, Merck Millipore, Darmstadt, Germany). Subsequently, the membranes were blocked for 1 h at room temperature in blocking solution (1× TBS supplemented with 0.2% Tween-20 and 5% skim milk) and washed three times with washing solution (1× TBS containing 0.2% Tween-20). Immunoblotting was performed overnight at 4°C with anti-p-CREB (1:1000), anti-CREB (1:1000), anti-MAO-A (1:1000), anti-SERT (1:2000), and anti-TPH2 (1:1000) antibodies. The membranes were subsequently washed five times and incubated with diluted horseradish peroxidase (HRP)-conjugated anti-rabbit IgG secondary antibodies (1:5000) for 1 h at room temperature. The detection was performed by using a chemiluminescence detection kit (Merck Millipore, Darmstadt, Germany) in accordance with the manufacturer’s instructions.

### Statistical Analysis

The data are presented as the SEM and statistically evaluated by Student’s *t*-test or ANOVA using GraphPad Prism (GraphPad Inc., San Diego, CA, United States) software program. The differences between the groups were assessed by using Duncan’s multiple range tests. A value of *P* < 0.05 was considered statistically significant.

## Results

### Standardization of VBLW

We identified the flavonoids chlorogenic acid, orientin, and isoorientin as three of the major constituents of VBLW; the retention times were consistent with standard compounds. Indeed, the retention times of chlorogenic acid, orientin, and isoorientin were typically detected at approximately 20.55, 32.56, and 34.09 min, respectively, in VBLW (**Figure 1**). The mean content of chlorogenic acid, orientin, and isoorientin in VBLW was approximately 7.06 mg/g, 4.87 mg/g, and 8.17 mg/g, respectively.

### Neuroprotective Effects of VBLW on CORT- and H_2_O_2_-Induced Cytotoxicity in SH-SY5Y Cells

**FIGURE 2:**
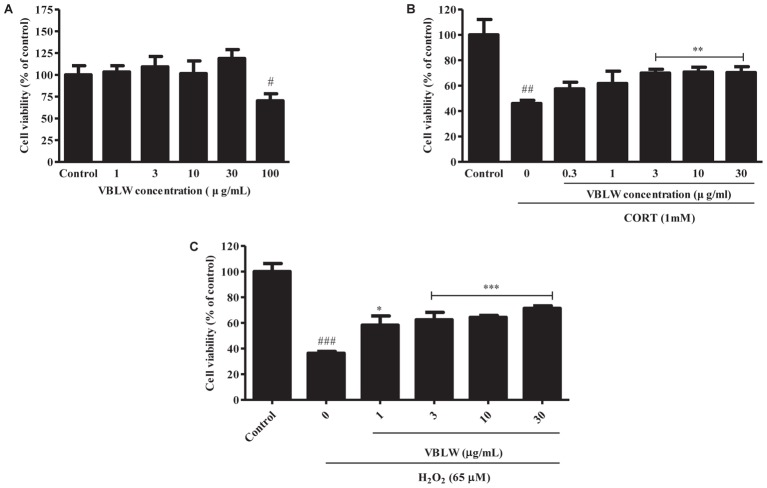
The effects of VBLW on cell proliferation and CORT- and H_2_O_2_-induced cytotoxicity in SH-SY5Y cells. **(A)** Cell viability was determined by using MTT assay with various concentrations of VBLW for 24 h. **(B,C)** SH-SY5Y cells were treated with CORT (1 mM) or H_2_O_2_ (65 μM) for 24 h in the absence or presence of VBLW at the indicated concentrations 2 h prior to CORT or H_2_O_2_ treatment and cell viability was measured by MTT assay. CORT, corticosterone; H_2_O_2_, hydrogen peroxide; MTT, 3-(4,5-dimethylthiazol-2-yl)-2,5-diphenyltetrazolium; VBLW, *Vaccinium bracteatum* leaves water extract. The values are expressed as the mean ± standard error of the mean (*n* = 3). ^#^*P* < 0.05, ^##^*P* < 0.01, and ^###^*P* < 0.001 compared with the control group; ^∗^*P* < 0.05, ^∗∗^*P* < 0.01, and ^∗∗∗^*P* < 0.001 compared with the CORT or H_2_O_2_ group.

To determine the protective effect of VBLW on CORT- and H_2_O_2_-induced cytotoxicity in SH-SY5Y cells, we measured the cytotoxicity of various concentrations of VBLW by using the MTT assay (**Figure 2A**). The results indicated that 1, 3, 10, and 30 μg/mL VBLW did not alter the viability of SH-SY5Y cells (*P* > 0.05). As shown in **Figure 2B**, 1 mM CORT induced a significant reduction in the cell viability to 45.98 ± 1.39% (^##^*P* < 0.01). At 0.3, 1, 3, 10, and 30 μg/mL, VBLW suppressed CORT-induced cytotoxicity to 57.46 ± 2.96%, 61.79 ± 5.38%, 69.98 ± 1.63%, 70.80 ± 2.68%, and 70.31 ± 3.28%, respectively (**Figure 2B**). Moreover, at 1, 3, 10, and 30 μg/mL, the H_2_O_2_-induced oxidative stress was inhibited to 58.24 ± 3.59%, 62.42 ± 1.64%, 64.38 ± 1.07%, and 71.37 ± 1.17%, respectively (^∗^*P* < 0.05 or ^∗∗∗^*P* < 0.001) (**Figure 2C**). Interestingly, 3, 10, and 30 μg/mL VBLW significantly reduced CORT- and H_2_O_2_-induced cytotoxicity in a dose-dependent manner (^∗∗^*P* < 0.01 and ^∗∗∗^*P* < 0.001, respectively). These results indicated that VBLW exerted significant neuroprotective effects against CORT-induced cytotoxicity, as well as H_2_O_2_-induced oxidative stress in SH-SY5Y cells.

### Effects of VBLW on PI3K/Akt/mTOR and PKA/ERKs Pathways in CORT-Induced Cytotoxicity in SH-SY5Y Cells

**FIGURE 3:**
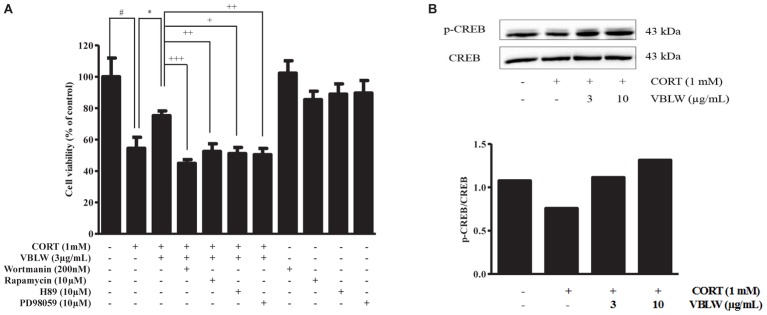
The effects of VBLW on PI3K/Akt/mTOR, PKA/ERKs, and CREB signaling pathways of CORT-induced cytotoxicity in SH-SY5Y cells. **(A)** SH-SY5Y cells were treated with 1 mM CORT for 24 h in the absence or presence of VBLW (3 μg/mL) treatment 2 h prior to CORT treatment. Wortmannin (200 nM), rapamycin (10 μM), H89 (10 μM), or PD98059 (10 μM) were treated 1 h prior to VBLW administration. The cell viability was determined by using the MTT assay. The values are expressed as the mean ± standard error of the mean (*n* = 3). ^#^*P* < 0.05 compared with the control group; ^∗^*P* < 0.05 compared with the CORT group; ^+^*P* < 0.05, ^++^*P* < 0.01, and ^+++^*P* < 0.001 compared with the CORT plus VBLW group. **(B)** SH-SY5Y cells were exposed to CORT (1 mM) for 12 h in the absence or presence of varying concentrations of VBLW 24 h prior to CORT treatment, and the cells were lysed. The protein expression levels of p-CREB/CREB were analyzed by western blotting. CORT, corticosterone; CREB, cyclic AMP-responsive element-binding protein; VBLW, *Vaccinium bracteatum* leaves water extract.

We then investigated the PI3K/mTOR/PKA/ERKs signaling pathways in the neuroprotective activity of VBLW exposed to CORT for 24 h. First, we pretreated the cells with wortmannin (200 nM), rapamycin (10 μM), H89 (10 μM), and PD98059 (10 μM), which are specific inhibitors of PI3K, mTOR, PKA, and ERKs, respectively. Subsequently, VBLW was added 2 h prior to CORT treatment for 24 h, and cell viability was determined by using the MTT assay. As shown in **Figure 3A**, CORT significantly decreased cell viability (54.45 ± 4.04%, ^#^*P* < 0.05) compared with the control group, whereas VBLW (3 μg/mL) increased the cell viability (75.21 ± 1.75%, ^∗^*P* < 0.05) compared with the CORT group. This improvement was blocked by wortmannin (44.84 ± 1.40%, ^+++^*P* < 0.001), rapamycin (51.08 ± 2.27%, ^++^*P* < 0.01), H89 (52.46 ± 2.82%, ^+^*P* < 0.05), and PD98059 (50.34 ± 2.31%, ^++^*P* < 0.01). However, individually, wortmannin, rapamycin, H89, and PD98059 showed no effect on SH-SY5Y cell viability.

We subsequently investigated which protein expression levels of CREB were involved in the neuroprotective effects of VBLW. The cells were treated with VBLW 24 h prior to CORT treatment, exposed to CORT (1 mM) for 12 h, and analyzed by using western blotting. The data showed that CORT decreased CREB phosphorylation, which was attenuated by VBLW treatment at 3 or 10 μg/mL (**Figure 3B**). These results suggested that VBLW exerted neuroprotective activity through the PKA/ERKs, PI3K/Akt, mTOR, and CREB signaling pathways.

### Effects of VBLW on Body Weight, Food Intake, and Organ Weight in CRS Mice

We investigated the antidepressant-like effects of VBLW (100 and 200 mg/kg/day, *p.o.*) in CRS mice for 21 days. The body weight, food intake, and organs weights of each group are shown in **Table 1**. After 21 days, the CRS group had a significantly reduced body weight gain compared with the control group (^###^*P* < 0.001). However, the final body weight gains of the VBLW 100 (30.7 ± 0.6 g) and VBLW 200 (31.1 ± 0.3 g) groups were not significantly different to the untreated CRS group. The food intake was not significantly different between the CRS and the VBLW groups. Therefore, the organ and body weights were not significantly altered in the VBLW-treated group compared with the CRS group (*P* > 0.05). Furthermore, we investigated the effects of VBLW on the serum levels of GOT, GPT and BUN in the CRS-exposed mice. VBLW alleviated two common markers for live damages, GOT and GPT, which were significantly increased by CRS-expose. In addition, the levels of BUN were significantly changed in CRS-exposed mice compared with that in the control group, while treatment with VBLW were not significantly changed compared to the levels in the CRS group. The liver and kidney damage markers, GOT, GPT and BUN, were not increased in the VBLW-administered groups (**Table 1**).

**Table 1:** Effects of VBLW on body weight, food intake, organ weights and blood parameter in CRS mice.

		CRS			
	Control	Vehicle	EO 10	VBLW100	VBLW200
Initial body weight (g)	27.0 1.0	27.0 1.0	27.0 1.5	27.0 1.3	27.0 1.2
Final body weight (g)	34.9 0.3	30.1 0.3###	32.1 0.2	30.7 0.6	31.1 0.3
Body weight gain (g)	7.9 0.3	3.1 0.5##	5.1 0.3	4.5 0.6	4.1 0.4
Food intake (g/day)	4.7 0.0	4.7 0.1	5.1 0.1	5.3 0.1	4.7 0.1
FER^1^)	1.7 0.1	0.7 0.1##	0.4 0.1	0.8 0.1	0.9 0.1
**Organ weight**					
Kidney (g)	0.59 0.01	0.56 0.02	0.55 0.01	0.56 0.02	0.55 0.01
Liver (g)	1.74 0.02	1.52 0.02 #	1.62 0.03	1.66 0.04	1.64 0.03
Spleen (g)	0.12 0.00	0.10 0.00 #	0.10 0.00	0.10 0.00	0.10 0.00
**Blood parameter**					
GOT (U/L)	51.00 1.13	81.43 6.30	78.67 2.30	72.75 11.31	69.33 1.56
GPT (U/L)	25.33 0.94	35.60 0.76#	37.67 0.87	37.40 1.95	32.83 0.49
BUN (mg/dL)	19.10 0.35	26.00 0.98#	19.08 0.34*	26.36 0.76	22.42 0.38

*^*1*^FER (Food efficiency ratio) = body weight gain/food intake. The values are expressed as mean ± standard deviation of the mean (*n* = 5). ^#^*P* < 0.05, ^##^*P* < 0.01 and ^###^*P* < 0.001 compared with control group; ^∗^*P* < 0.05 compared with CRS group.*

### Effects of VBLW on the Immobility Time in FST in CRS Mice

**FIGURE 4:**
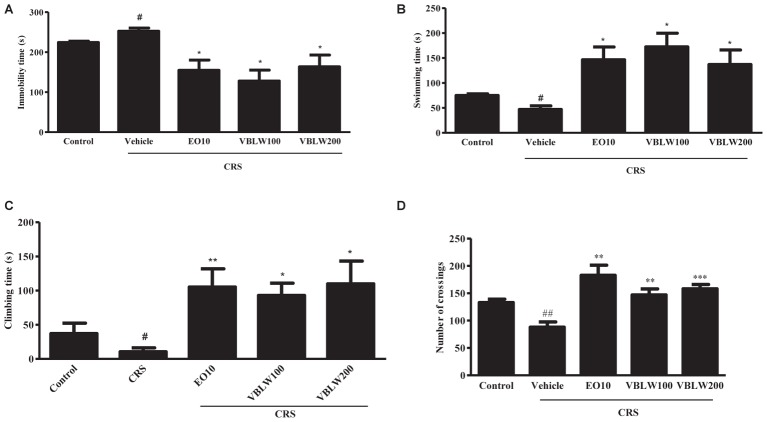
The effects of VBLW in the FST and OFT in CRS-induced mice. The effects of VBLW on the duration of immobility **(A)**, swimming **(B)**, and climbing **(C)** behaviors in the FST in CRS mice. The duration of immobility, swimming, and climbing was scored during the final 5 min of a 6-min test. **(D)** The effects of VBLW on locomotor activity (the number of crossings) in the OFT in CRS mice. Locomotor activity was over during a 5 min session. CRS, chronic restraint stress; EO10, escitalopram oxalate 10 mg/kg; VBLW100, *Vaccinium bracteatum* leaves water extract 100 mg/kg; VBLW200, *Vaccinium bracteatum* leaves water extract 200 mg/kg. The values are expressed as the mean ± standard error of the mean (*n* = 5). ^#^*P* < 0.05 and ^##^*P* < 0.01 compared with the control group; ^∗^*P* < 0.05, ^∗∗^*P* < 0.01, and ^∗∗∗^*P* < 0.001 compared with the CRS group.

CRS-exposed mice showed significant changes in the duration of immobility, swimming, and climbing behavior compared with the control group (**Figure 4**) (^#^*P* < 0.05, respectively). However, VBLW (100 and 200 mg/kg) significantly induced a decrease in the immobility time compared with the CRS group (**Figure 4A**) (^∗^*P* < 0.05, respectively). As shown in **Figures 4B,C**, treatment with VBLW (100 and 200 mg/kg) resulted in a significant increase in the duration of swimming (^∗^*P* < 0.05) and climbing (^∗^*P* < 0.05) compared with the CRS group. EO (10 mg/kg; positive control) significantly reduced the immobility time (^∗^*P* < 0.05) and ameliorated the swimming (^∗^*P* < 0.05) and climbing (^∗∗^*P* < 0.01) behaviors.

### Effects of VBLW on the Locomotor Activity in CRS Mice

The effects of EO (10 mg/kg) and VBLW (100 and 200 mg/kg) on the locomotor activity using OFT are shown in **Figure 4D**. CRS-exposed mice significantly decreased the number of crossings compared with the control group (^##^*P* < 0.01). However, treatment with VBLW 100 mg/kg (^∗∗^*P* < 0.01) and VBLW 200 mg/kg (^∗∗∗^*P* < 0.001) displayed a significant increase in the locomotor activity compared with the CRS group.

### Effects of VBLW on HPA Axis Changes in CRS Mice

**FIGURE 5:**
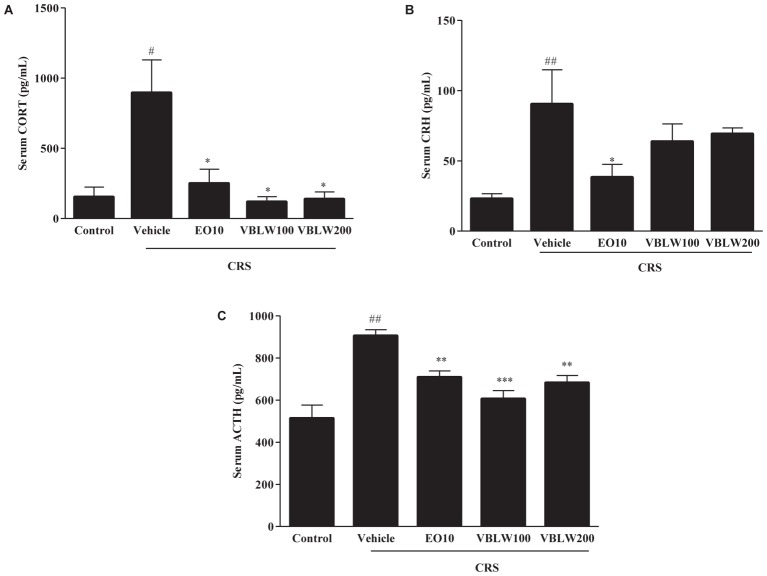
**(A–C)** The effects of VBLW on serum CORT, CRH, and ACTH concentrations in CRS-induced mice. CRS, chronic restraint stress; EO10, escitalopram oxalate 10 mg/kg; VBLW100, *Vaccinium bracteatum* leaves water extract 100 mg/kg; VBLW200, *Vaccinium bracteatum* leaves water extract 200 mg/kg. The values are expressed as the mean ± standard error of the mean (*n* = 5). ^#^*P* < 0.05, ^##^*P* < 0.01, and ^###^*P* < 0.001 compared with the control group; ^∗^*P* < 0.05, ^∗∗^*P* < 0.01, and ^∗∗∗^*P* < 0.001 compared with the CRS group.

We investigated the effect of VBLW on the serum CORT, CRH, and ACTH levels in CRS mice. As shown in **Figure 5**, the CRS-exposed mice exhibited a significantly elevated level of serum CORT (^#^*P* < 0.05), CRH (^##^*P* < 0.01), and ACTH (^##^*P* < 0.01) compared with the control group. In contrast, VBLW (100 and 200 mg/kg) effectively decreased the serum CORT (^∗^*P* < 0.05, respectively), CRH (*P* > 0.05), and ACTH (^∗∗^*P* < 0.01 and ^∗∗∗^*P* < 0.001, respectively) levels compared with the CRS group. EO (10 mg/kg) treatment also markedly decreased the levels of CORT (^∗^*P* < 0.05), CRH (^∗^*P* < 0.05), and ACTH (^∗∗^*P* < 0.01), respectively.

### Effects of VBLW on Monoamine Neurotransmitter Levels in CRS Mice

**FIGURE 6:**
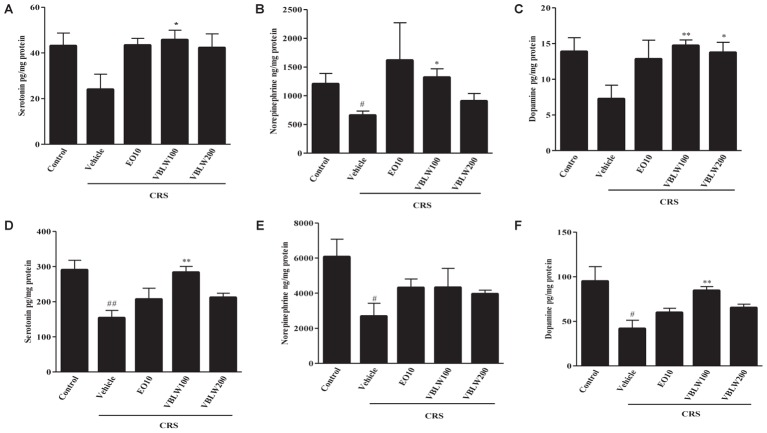
The effects of VBLW on monoamine neurotransmitter concentrations in hippocampus and PFC in CRS-induced mice: **(A)** Serotonin, **(B)** norepinephrine, and **(C)** dopamine levels in the hippocampus and **(D)** serotonin, **(E)** norepinephrine, and **(F)** dopamine in the PFC. CRS, chronic restraint stress; EO10, escitalopram oxalate 10 mg/kg; PFC, prefrontal cortex; VBLW100, *Vaccinium bracteatum* leaves water extract 100 mg/kg; VBLW200, *Vaccinium bracteatum* leaves water extract 200 mg/kg. The values are expressed as the mean ± standard error of the mean (*n* = 5). ^#^*P* < 0.05 and ^##^*P* < 0.01 compared with the control group; ^∗^*P* < 0.05 and ^∗∗^*P* < 0.01 compared with the CRS group.

As shown in **Figures 6A,D**, repeated CRS for 21 days significantly decreased the serotonin levels in the hippocampus (*P* > 0.05) and the PFC (^##^*P* < 0.01) compared with those in the control group. However, the daily administration of VBLW (100 mg/kg) significantly elevated the serotonin levels in the hippocampus (^∗^*P* < 0.05) and the PFC (^∗∗^*P* < 0.01) compared with those in the CRS group, but not in the VBLW (200 mg/kg).

As shown in **Figures 6B,E**, when mice were exposed to CRS for 21 days, the norepinephrine levels in the hippocampus (^#^*P* < 0.05) and the PFC (^#^*P* < 0.05) were lower than those observed in the control group. In the PFC, EO (10 mg/kg) and VBLW (100 and 200 mg/kg) did not significantly increase the norepinephrine levels compared with the CRS group (*P* > 0.05). However, norepinephrine levels in the hippocampus was significantly increased by the treatment of VBLW (100 mg/kg) (^∗^*P* < 0.05).

Moreover, when mice were exposed to CRS for 21 days, the dopamine levels in the hippocampus (*P* > 0.05) and the PFC (^#^*P* < 0.05) were lower than those in the control group (**Figures 6C,F**). However, the administration of VBLW (100 mg/kg) significantly elevated the dopamine levels in the hippocampus and the PFC compared with those in the CRS group (^∗∗^*P* < 0.01, respectively). EO (10 mg/kg) and VBLW (200 mg/kg) did not result in significant changes in the levels of dopamine in the PFC (*P* > 0.05).

### Effects of VBLW on TPH2, SERT, and MAO-A Protein Levels in CRS Mice

**FIGURE 7:**
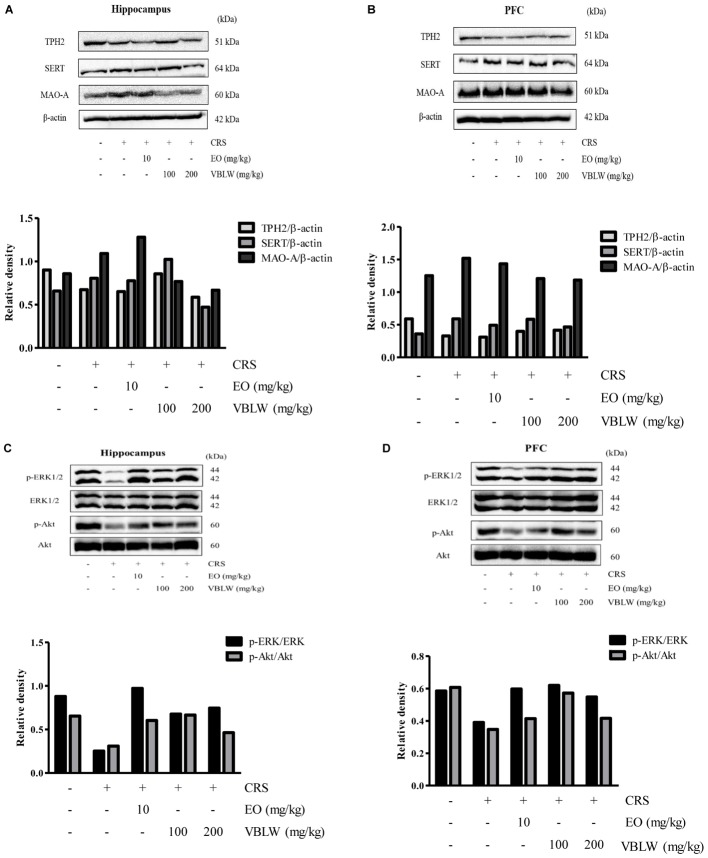
The effects of VBLW on TPH2, SERT, MAO-A, ERK1/2, and Akt protein expression in the hippocampus **(A,C)** and the PFC **(B,D)** in CRS-induced mice. Akt, protein kinase B; ERKs, extracellular signal-regulated kinases; MAO-A, monoamine oxidase A; SERT, serotonin transporter; TPH2, Tryptophan hydroxylase 2; VBLW, *Vaccinium bracteatum* leaves water extract.

Serotonin turnover systems were associated with key enzymes involved in biosynthesis, reuptake, and degradation, such as TPH2, SERT, and MAO-A. The expression of SERT, TPH2, and MAO-A were analyzed by using western blotting. As shown in **Figures 7A,B**, the CRS exposed mice reduced TPH2 in the hippocampus and the PFC as compared with the control group, whereas treatment with VBLW caused an increase in the TPH2 protein level in the hippocampus and the PFC compared with the CRS group. In addition, CRS-exposed mice increased MAO-A and SERT compared with the control group, whereas the treatment with VBLW caused a decrease in MAO-A and SERT in the hippocampus and the PFC compared with the CRS group.

### Effects of VBLW on ERK1/2 and Akt Phosphorylation in CRS Mice

We next investigated whether the ERK1/2 and Akt pathways within the hippocampus and the PFC were involved in the antidepressant-like effects of VBLW. As shown in **Figures 7C,D**, the CRS mice reduced the phosphorylation of ERK1/2 and Akt signaling in the hippocampus and the PFC, whereas treatment with VBLW (100 and 200 mg/kg) normalized these levels. Similarly, the phosphorylation of ERK1/2 and Akt signaling in the hippocampus and the PFC was increased by the treatment with EO (10 mg/kg) compared with the CRS group.

### Effects of the Organic Solvent Fractions Obtained From VBLW on CORT-Induced Cytotoxicity in SH-SY5Y Cells

**FIGURE 8:**
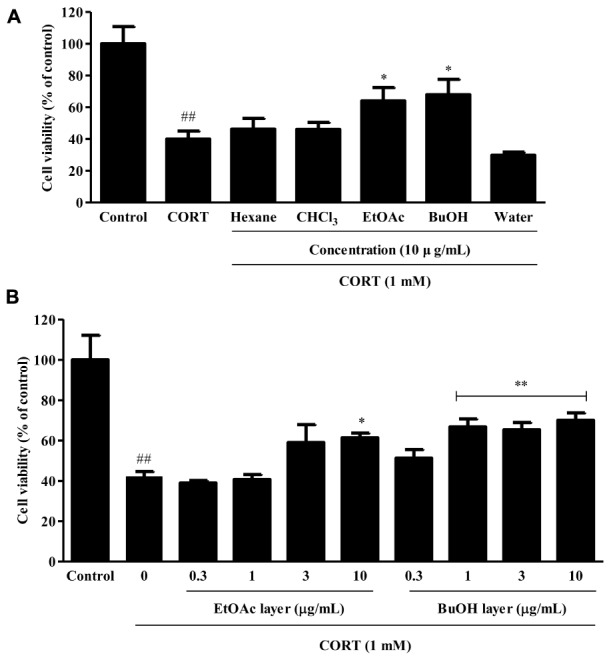
The effects of the organic solvent fractions obtained from VBLW on CORT-induced cytotoxicity in SH-SY5Y cells. **(A)** SH-SY5Y cells were treated with CORT (1 mM) for 24 h in the absence or presence of the organic solvent fractions at concentration 10 μg/mL 2 h prior to CORT treatment. **(B)** The EtOAc and BuOH fractions were treated at various concentrations (0.3–10 μg/mL) 2 h prior to CORT treatment. Cell viability was measured by MTT assay. CORT, corticosterone; CHCl_3_, chloroform; EtOAc, ethyl acetate; BuOH, *n*-butanol; MTT, 3-(4, 5-dimethylthiazol-2-yl)-2,5-diphenyltetrazolium. The values are expressed as the mean ± standard error of the mean (*n* = 3). ^##^*P* < 0.01 compared with the control group; ^∗^*P* < 0.05 and ^∗∗^*P* < 0.01 compared with the CORT group.

To examine the protection of organic solvent fractions obtained from VBLW on CORT-induced cytotoxicity in SH-SY5Y cells, the cytotoxicity of the organic solvent fractions of VBLW at 10 μg/mL were measured by using the MTT assay. As shown in **Figure 8A**, the protective effects of the EtOAc and BuOH solvent fractions against CORT-induced cytotoxicity were approximately 64.08 ± 4.85% and 67.97 ± 5.58%, respectively (^∗^*P* < 0.05). However, there were no significant differences in the cell viability between the hexane, CHCl_3_, and water fractions. At 0.3, 1, 3, and 10 μg/mL BuOH, CORT-induced cytotoxicity to 51.25 ± 2.49%, 66.72 ± 2.68%, 65.30 ± 2.08%, and 69.99 ± 2.14%, respectively (^∗∗^*P* < 0.01) (**Figure 8B**). These protective effects were achieved at a concentration that did not affect cell viability (*P* > 0.05) (data not shown). These results indicated that, among the five isolated organic solvent fractions, the BuOH solvent fraction significantly reduced CORT-induced cytotoxicity in SH-SY5Y cells.

## Discussion

Major depressive disorder is the most common depressive illness, which is characterized by clinical symptoms such as low mood, marked loss of interest, fatigue, worthlessness, insomnia, and suicidal ideation (American Psychiatric Association, 2013). Several types of antidepressant drugs are widely available, including SSRIs, tricyclic antidepressants (TCAs), serotonin-noradrenaline reuptake inhibitors (SNRIs), and monoamine oxidase inhibitors (MAOIs). Recent studies have found traditional herbal medicine to be effective complementary and alternative therapies for depression (Qureshi and Al-Bedah, 2013; Huang et al., 2018) that are expected to induce less severe side effects (such as weight gain, insomnia, constipation, cardiovascular, and metabolic disorders) (Henderson, 2008) compared with traditional antidepressant drugs. Traditional herbal medicine (e.g., *Ginkgo biloba* and *Gastrodia elata*) have demonstrated beneficial antidepressant-like effects in attenuating side-effects (Atmaca et al., 2005; Chen et al., 2008).

In a previous study, we demonstrated the antidepressant-like effects of *V. bracteatum* fruit extract might be the regulation of monoaminergic systems and serum CORT that was possibly associated with the neuroprotective effects through the ERKs/Akt/mTOR/CREB signaling pathways as well as the antagonism of the 5-HT_2A_ receptor (Oh et al., 2018). In some previous papers, various parts of the herbal plants, such as the leaves, fruits, bark, and root, have been used as food and herbal medicine; different parts may have different effects (Singab et al., 2005; Kai et al., 2014). In the present study, we confirmed that the neuroprotective effects of VBLW against the CORT-induced cytotoxicity in SH-SY5Y cells. To mimic the *in vitro* conditions of depression, reflected by an elevated CORT level via the activity of the HPA axis, we exposed SH-SY5Y cells to a high concentration of CORT (1 mM). We found that VBLW significantly reduced CORT-induced cell cytotoxicity in SH-SY5Y cells and significantly reduced H_2_O_2_-induced oxidative stress in a dose-dependent manner (**Figure 2**). Previous research has suggested several mechanisms, including various pathogeneses for depression (e.g., hyper-cortisolemia, monoamine deficiency, and increased levels of inflammatory cytokines), have been proposed (Belmaker and Agam, 2008). In the past few years, neuroprotection has constituted an important mechanism of neuropsychiatric drug actions; these processes are found in the structure and function of neural cells and in animal models of depression (Campos et al., 2017). In addition, oxidative stress has been investigated as a contributing factor in the pathogenesis of MDD (Ng et al., 2008). The neuroprotective mechanism against CORT-induced injury is mediated though the inhibition of oxidative and endoplasmic reticulum stress-mediated apoptosis.

Neuronal survival requires the ERKs/PI3K-Akt-mTOR and CREB signaling pathways for activation of this process. In the present study, we first examined the effects of the specific inhibitors wortmannin (PI3K inhibitor), rapamycin (mTOR inhibitor), H89 (PKA inhibitor), and PD98059 (ERK inhibitor) on the neuroprotective effects of VBLW. The results showed that pretreatment with specific inhibitors blocked VBLW-mediated neuroprotection against CORT-induced cytotoxicity, which indicated that the neuroprotective effects of VBLW occurred through the activation of the PI3K/Akt, mTOR, and ERKs signaling pathways. CREB plays a key role in the regulation of cellular survival and mediates the responses to neurotrophic factors; the inhibition of CREB activity induces apoptosis (François et al., 2000). Previously, other reports have shown that ERK activates the transcription factor CREB and that antidepressants also exhibited neuroprotective activity, possibly through the PKA-ERK, PI3K-Akt, and CREB signaling pathways (Lim et al., 2008). To determine the mechanisms of the neuroprotective effects against CORT-induced cytotoxicity, the actions on the CREB pathways were examined. We showed that phosphorylated CREB levels were reduced by the treatment with CORT, whereas the combined treatment of CORT and VBLW reversed this decrease (**Figure 3B**). Collectively, these results showed that VBLW increased CREB phosphorylation in CORT-induced cell injury, which was mediated through the ERK/Akt/mTOR signaling pathways.

Chronic stress over a long period can cause depression in humans. The CRS procedure is an animal model to mimic the development of clinical depression, inducing changes in body weight, behavior (such as immobility increase and locomotor activity deficits), hormones (such as glucocorticoid levels), and monoamine neurotransmitters (Mahar et al., 2014). In contrast, the administration of antidepressant drugs reverses animal behavior and hormonal changes induced by depression (Park et al., 2014; Li et al., 2015). The results of the present study showed that CRS mice exhibited depression-like behavior, which was characterized by decreased body weight, food efficiency ratio, locomotor activity deficit, and increased immobility time. However, the treatment with VBLW significantly controlled the depressive behavior through a decrease in immobility time (*P* < 0.05) and an increase in locomotor activity (*P* < 0.01 and *P* < 0.001). Similarly, EO administration significantly changes in the immobility and locomotor activity (*P* < 0.05 and *P* < 0.01, respectively). In addition, previous studies demonstrated that Antidepressants (SSRI, e.g. fluoxetine and imipramine) cause a decrease in immobility time in the FST and increase in locomotor activity in mice in the OFT (Potdar and Kibile, 2011).

In general, chronic stress is mediated by hyperactivity of the HPA axis in patients with depressive-like symptoms (Swaab et al., 2005). Previous studies have suggested that CRS is known to upregulate the stress hormones through hyperactivation of the HPA axis, which alters the catecholaminergic and monoaminergic systems. In the present study, we found that CRS mice elevated serum CORT levels through induction of the hyperactivity of the HPA axis, which was supported by the increased levels of CRH and ACTH. The administration of VBLW acted as antidepressant-like drug in CRS mice, as evidenced by normalization of the HPA axis and decreased serum CORT levels.

Furthermore, the interaction of the HPA axis and the monoamine system in brain regions, particularly in the PFC and the hippocampus, are important in depressive disorders, which leads to the depletion of monoamine neurotransmitters (Belmaker and Agam, 2008; Hamon and Blier, 2013). The monoamine hypothesis has demonstrated that neurotransmitters (e.g., serotonin, dopamine, and norepinephrine) are critically involved in depression. In the present study, our findings demonstrated CRS exposure significantly decreased the levels of serotonin, dopamine, and norepinephrine in both the PFC and hippocampal regions (**Figure 6**). However, VBLW treatment elevated these neurotransmitters in both the PFC and the hippocampus. Antidepressants, such as SSRIs and MAOIs, were increased the level of monoamine neurotransmitters in synaptic cleft. These leads to regulation of postsynaptic G protein coupled receptors, which mediate to a second messenger systems including PKA-ERK-CREB and PI3K-Akt pathways (Krishnan and Nestler, 2008). The ERK/Akt pathway plays an important role in the transcriptional and translational activation in neuronal survival and neuroplasticity in depression. Chronic stress exposure reduced the phosphorylation of ERKs and Akt in the hippocampus and PFC (Chandran et al., 2013). Furthermore, studies also suggested that VBLW normalized the CRS-induced depressive-like behavior by alternative mechanisms, such as the ERK and Akt signaling pathways (**Figures 7C,D**). Accordingly, as our results indicated that VBLW may be mediated by the action of various post-synaptic receptors, these mechanisms need to be studied further.

Moreover, earlier studies reported that the selective inhibition of MAO was involved in antidepressant-like effects (Lin et al., 2005). MAO has two isoforms, MAO-A and MAO-B, which have different distributions; MAO-A is mainly, but not exclusively, located in brain neurons (particularly in the hippocampus and cortex), whereas MAO-B is preferentially placed in glia and astrocytes (Shih and Chen, 2004). MAO-A act to degrade monoamine neurotransmitters, which primarily deaminate serotonin, dopamine, and norepinephrine (Youdim et al., 2006). In addition, previous studies demonstrated that chronic stress increases MAO-A protein expression (Duncan et al., 2012). The results of this study support that VBLW inhibited the protein level of MAO-A in the hippocampus and PFC of CRS-exposed mice (**Figure 7**), which was associated with changes in monoamine neurotransmitter levels.

The study of (Cui and Zheng, 2016) reported that the inhibited of SERT also plays an important role in the improvement of depressive disorders. SERT, are known as the serotonergic transporter, transports released serotonin from the synaptic cleft into the presynaptic neuron terminals and is involved pharmacological targets, such as SSRIs (Baudry et al., 2010). SSRIs are second-generation antidepressants commonly used to treat both depression and anxiety disorders; they block the reuptake of serotonin and result in an increased concentration of serotonin in the synaptic cleft. Chronic stress induced CORT release and leads to the upregulation of SERT protein levels in the brain region (e.g., dorsal raphe nucleus, hippocampus, and frontal cortex) (Filipenko et al., 2002; Gardner et al., 2009; Zhang et al., 2012). In the present study, we demonstrated that CRS exposure increased SERT protein levels in both brain regions, whereas treatment with VBLW significantly reduced SERT expression in both brain regions (**Figure 7**). These results suggested that the antidepressant-like effects of VBLW were possibly mediated by the inhibition of SERT in the hippocampus and the PFC.

In the present study, the results were confirmed through the decreased TPH2 protein level in CRS mice, whereas treatment with VBLW exerted a weak regulatory action on the inhibition of TPH2 expression in both brain regions (**Figure 7**). TPH, the rate-limiting enzyme in 5-HT synthesis, has two known isoforms, including a neuron-specific TPH2 isoform (Chen and Miller, 2013). Recent studies have indicated that TPH2 activity is significantly decreased after induction of a stress-depressive model in brain tissue (Chen et al., 2017). Thus, the activation of TPH2 may open new perspectives for the treatment of neurological and psychiatric disorders through alterations of the serotonin levels in the brain (Matthes et al., 2010).

A flavonoid compound found in *V. bracteatum*, such as orientin, has been shown to exert an antioxidant effect (Liu et al., 2015). Together with previous reports, which demonstrated the potential antioxidant effects of flavonoids in many natural herbs, these flavonoid compounds, such as orientin, are known to exert antidepressant-like effects. Our results demonstrated that orientin content in VBLW was approximately 4.87 mg/g extract (**Figure 1**). Moreover, 1, 3, 5, 10, and 30 μg/mL VBLW was equivalent to an orientin content of 4.9, 14.6, 24.3, 48.7, and 146.1 ng/mL, respectively. We further demonstrated that orientin did not have a significant neuroprotective effect against CORT-induced cytotoxicity (**Supplementary Figure S1**). In **Figure 1**, several peaks were found that suggested the involvement of different active compounds. The results showed that it contained many different active constituents, such as chlorogenic acid and isoorientin. Previous studies reported that flavonoids contributed to the pharmacological activities and showed antidepressant effects, as well as antioxidant effects (Hritcu et al., 2017). Chlorogenic acid is a polyphenol found in plant families, which suggests that the antidepressant-like effects are mediated through decrease immobility time in the tail suspension test and the FST (Park et al., 2010). However, further studies are required to determine the functional active constituents that are involved in the antidepressant effects of VBLW.

## Conclusion

**FIGURE 9:**
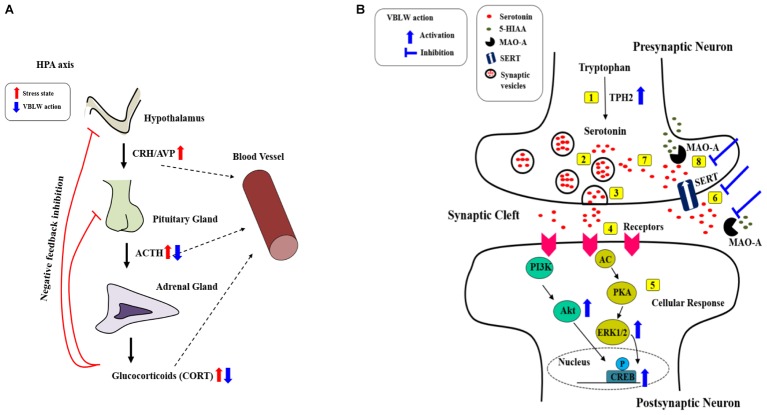
Possible antidepressant mechanisms of action of VBLW. **(A)** HPA axis. **(B)** Serotonergic systems at the synapse: [1] Serotonin synthesis; [2] serotonin into vesicles; [3] vesicles releases into the synaptic cleft; [4] serotonin binds to receptor; [5] serotonin binding receptor initiates cellular signaling pathways; [6] the reuptake of serotonin in the synaptic cleft into presynaptic neurons occurs; [7] serotonin recycling into vesicles; or [8] MAO breaks down serotonin. AC, adenylyl cyclase; ACTH, adrenocorticotropic hormone; Akt, protein kinase B; AVP, Arginine-vasopressin; CORT, corticosterone; CREB, cyclic AMP response element binding protein; CRH, corticotropin-releasing hormone; ERK1/2, extracellular signal–regulated kinases1/2; 5-HIAA, 5-hydroxyindoleacetic acid; MAO-A, monoamine oxidase A; PKA, protein kinase A; PI3K, phosphatidylinositide 3-kinases; SERT, serotonin transporter; TPH2, Tryptophan hydroxylase 2; VBLW, *Vaccinium bracteatum* leaves water extract.

Our results demonstrated that VBLW exerted antidepressant-like effects by the regulation of neuroprotective activity through CREB signaling pathways, including the PKA/ERKs/Akt pathways. Moreover, VBLW induced antidepressant-like behavior changes in the FST and OFT in CRS-exposed mice. The effects of VBLW were possibly mediated by the biosynthesis of serotonin, the normalization of SERT and MAO-A, increased monoamine neurotransmitters and ERK/Akt phosphorylation, and prevention of HPA axis dysfunction, which may be the molecular and cellular mechanisms underlying the antidepressant-like effects of VBLW on the CRS-exposed mice (**Figure 9**). Furthermore, we demonstrated that the BuOH fractions obtained from VBLW showed the highest neuroprotective activity *in vitro*. Therefore, further studies are necessary to define the bioactive compounds that regulate the antidepressant-like effects of VBLW.

## Author Contributions

D-RO and J-SY carried out all the assays. YK, M-AJ, DB, K-NO, J-AH, AJ, JS, and JK carried out experiments. HK, HL, SL, and E-jC carried out HPLC analysis. YRK, SC, and B-JL participated in the design of the study and performed the statistical analysis. CC conceived of the study and participated in its design and coordination and helped to draft the manuscript.

## Conflict of Interest Statement

The authors declare that the research was conducted in the absence of any commercial or financial relationships that could be construed as a potential conflict of interest. The reviewer HS and handling Editor declared their shared affiliation.

## Supplementary Material

FIGURE S1 The effects of VBLW and orientin on CORT-induced cytotoxicity in SH-SY5Y cells. SH-SY5Y cells were treated with CORT (1 mM) for 24 h in the absence or presence of VBLW and orientin at the indicated concentrations 2 h prior to CORT treatment, and cell viability was measured by using the MTT assay. CORT, corticosterone; MTT, 3-(4,5-dimethylthiazol-2-yl)-2,5-diphenyltetrazolium; VBLW, *Vaccinium bracteatum* leaves water extract. The values are expressed as the mean ± standard error of the mean (*n* = 3). ^#^*P* < 0.05 compared with the control group; ^∗^*P* < 0.05 and ^∗∗^*P* < 0.01 compared with the CORT group.

## Abbreviations

ACTH: adrenocorticotropic hormone
ANOVA: one-way analysis of variance
Akt: protein kinase B
BCA: bicinchoninic acid assay
BDNF: brain-derived neurotrophic factor
BSA: bovine serum albumin
CORT: corticosterone
CREB: cyclic AMP-responsive element-binding protein
CRH: corticotropin releasing hormone
CRS: chronic restraint stress
DTT: dithiothreitol
ELISA: enzyme-linked immunosorbent assay
ERKs: downstream extracellular signal-regulated kinases
FBS: fetal bovine serum
FST: forced swim test
HPA: hypothalamic-pituitary-adrenal
HPLC: high-performance liquid chromatography
MAO-A: monoamine oxidase A
MDD: major depressive disorder
MEM: Minimum Essential Medium
mTOR: mammalian target of rapamycin
MTT: 3-(4,5-dimethylthiazol-2-yl)-2,5-diphenyltetrazolium
OD: optical density
OFT: open field test
PI3K: phosphatidylinositol 3-kinase
PKA: protein kinase A
PMSF: phenylmethylsulfonyl fluoride
PVDF: polyvinylidene difluoride
SEM: mean ± standard error of the mean
SERT: serotonin reuptake
TPH: tryptophan hydroxylase
VBL: *Vaccinium bracteatum* leaves
VBLW: *Vaccinium bracteatum* Thunb. leaves water extract
