# Supplementary figures and images for: Vaccinium bracteatum Leaf Extract Reverses Chronic Restraint Stress-Induced Depression-Like Behavior in Mice: Regulation of Hypothalamic-Pituitary-Adrenal Axis, Serotonin Turnover Systems, and ERK/Akt Phosphorylation
Source: Front Pharmacol. 2018 Jul 9;9:604. doi: 10.3389/fphar.2018.00604 (PMC6047486; doi:10.3389/fphar.2018.00604)

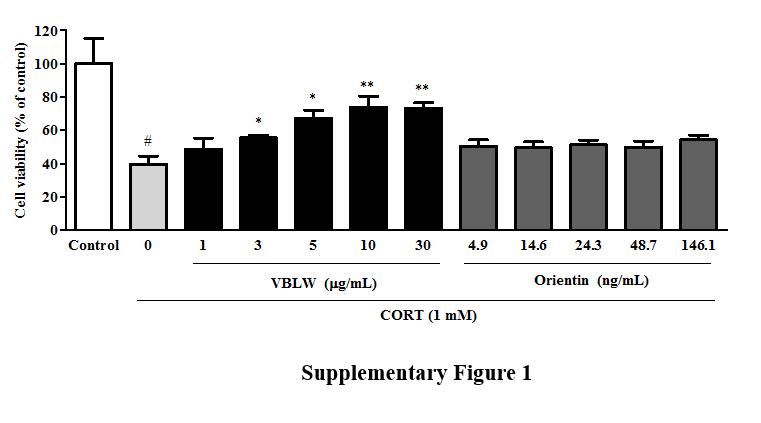

Supplement: FIGURE S1 — The effects of VBLW and orientin on CORT-induced cytotoxicity in SH-SY5Y cells. SH-SY5Y cells were treated with CORT (1 mM) for 24 h in the absence or presence of VBLW and orientin at the indicated concentrations 2 h prior to CORT treatment, and cell viability was measured by using the MTT assay. CORT, corticosterone; MTT, 3-(4,5-dimethylthiazol-2-yl)-2,5-diphenyltetrazolium; VBLW, Vaccinium bracteatum leaves water extract. The values are expressed as the mean ± standard error of the mean (n = 3). #P < 0.05 compared with the control group; ∗P < 0.05 and ∗∗P < 0.01 compared with the CORT group. [file Image_1.JPEG]
